# How to Improve the Biocompatibility of Peritoneal Dialysis Solutions (without Jeopardizing the Patient’s Health)

**DOI:** 10.3390/ijms22157955

**Published:** 2021-07-26

**Authors:** Mario Bonomini, Valentina Masola, Giuseppe Procino, Victor Zammit, José C. Divino-Filho, Arduino Arduini, Giovanni Gambaro

**Affiliations:** 1 Nephrology and Dialysis Unit, Department of Medicine, G. d’Annunzio University, Chieti-Pescara, SS. Annunziata Hospital, Via dei Vestini, 66013 Chieti, Italy; 2 Division of Nephrology and Dialysis, Department of Medicine, University of Verona, Piazzale A. Stefani 1, 37126 Verona, Italy; valentina.masola@unipd.it (V.M.); giovanni.gambaro@univr.it (G.G.); 3 Department of Biomedical Sciences, University of Padova, Viale G. Colombo 3, 35121 Padova, Italy; 4 Department of Biosciences, Biotechnologies and Biopharmaceutics, University of Bari, 70125 Bari, Italy; giuseppe.procino@uniba.it; 5 Translational & Experimental Medicine, Warwick Medical School, University of Warwick, Coventry CV4 7AL, UK; V.A.Zammit@warwick.ac.uk; 6 Division of Renal Medicine, CLINTEC, Karolinska Institutet, 17177 Stockholm, Sweden; jose.divino@ki.se; 7 Department of Research and Development, Iperboreal Pharma, 65100 Pescara, Italy; a.arduini@iperboreal.com

**Keywords:** peritoneal dialysis, glucose, L-carnitine, xylitol, alanyl-glutamine, solution, biocompatibility, PD fluid, peritoneal fibrosis

## Abstract

Peritoneal dialysis (PD) is an important, if underprescribed, modality for the treatment of patients with end-stage kidney disease. Among the barriers to its wider use are the deleterious effects of currently commercially available glucose-based PD solutions on the morphological integrity and function of the peritoneal membrane due to fibrosis. This is primarily driven by hyperglycaemia due to its effects, through multiple cytokine and transcription factor signalling—and their metabolic sequelae—on the synthesis of collagen and other extracellular membrane components. In this review, we outline these interactions and explore how novel PD solution formulations are aimed at utilizing this knowledge to minimise the complications associated with fibrosis, while maintaining adequate rates of ultrafiltration across the peritoneal membrane and preservation of patient urinary volumes. We discuss the development of a new generation of reduced-glucose PD solutions that employ a variety of osmotically active constituents and highlight the biochemical rationale underlying optimization of oxidative metabolism within the peritoneal membrane. They are aimed at achieving optimal clinical outcomes and improving the whole-body metabolic profile of patients, particularly those who are glucose-intolerant, insulin-resistant, or diabetic, and for whom daily exposure to high doses of glucose is contraindicated.

## 1. Introduction

The increasing worldwide number of patients suffering from end-stage kidney failure (ESKF) who require a chronic renal replacement therapy (RRT) represents a significant economic burden on health systems globally [1]. Among RRTs, peritoneal dialysis (PD) represents a well-established, cost-effective modality that can be delivered at home. It is based on the depuration of uremic blood through exchanges between peritoneal capillaries and a solution infused into the peritoneal cavity via an implanted catheter (dialytic exchange). PD solutions (dialysate, PD fluid) contain physiological concentrations of electrolytes, a buffer (lactate and/or bicarbonate) to correct uremic acidosis, and an osmotic agent (usually glucose) to induce water flow, and thereby ultrafiltration (UF), across the peritoneal capillaries to counteract water retention. After a specified dwell time, the effluent is drained out and fresh dialysate is reinfused into the abdomen. The dialytic exchange can be performed manually (continuous ambulatory PD; CAPD) or by employing a cycler (automated PD; APD), usually during the night.

**Figure 1:**
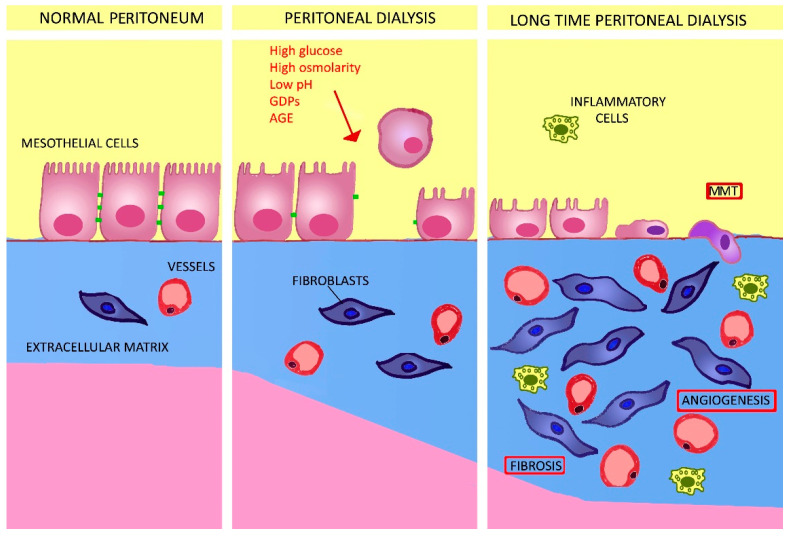
Schematic representation of a cross-section of the peritoneum during PD. The left-hand panel shows a normal peritoneal membrane. During PD, a series of deleterious factors injure the mesothelium (middle panel) with a consequent loss of the brush border, loss of cell-to-cell contact structure, and cell detachment from the basement membrane. Moreover, in response to cytokines and growth factors, fibroblasts are activated to myofibroblasts, which increase the secreted extracellular matrix. In long-term PD, there is significant damage to the mesothelial layer (right-hand panel), and mesothelial cells undergo MMT, thus contributing to the expansion of fibrosis. Inflammatory cells are also recruited and a neoangiogenic response is activated, leading to ultrafiltration failure. Abbreviations: GDPs: glucose-degradation products; AGE: advanced glycosylation end product; MMT: mesothelial-to-mesenchymal transition.

Compared with hemodialysis, PD offers a similar survival rate and is less expensive; in addition, it preserves residual kidney function better and removes solutes and fluid more gradually [2]. However, despite PD being a viable treatment for ESKF patients, it is still underprescribed [1]. This may be explained by major limitations in PD efficiency and sustainability [3], primarily due to infections including peritonitis and catheter patency. Consequently, PD may be abandoned after relatively short periods of treatment [4,5]. For patients on long-term PD, poor biocompatibility of the dialysis fluid (i.e., its capacity to preserve the natural anatomical and functional characteristics of the peritoneal membrane) is the major concern. Indeed, as detailed in the section below, prolonged exposure of the peritoneal membrane to the highly unphysiological composition of conventional PD fluids may cause neoangiogenesis, inflammation, and fibrosis (Figure 1) [6,7]. Such damage to the peritoneum is characterized by increased transport of low-molecular-weight solutes and a loss of UF capacity, eventually leading to UF failure [8].

Development of a more biocompatible, efficient dialysate for routine clinical use is of utmost importance for the future of PD. Many factors have been claimed [9] as contributors to the bioincompatibility of standard PD solutions, including their high glucose content, elevated levels of glucose-degradation products (GDPs) generated during heat sterilization of glucose-based solutions, acidic pH, high osmolarity, and use of a lactate buffer (Figure 1).

In this article, we review established and novel approaches aimed at improving the biocompatibility of PD solutions, and hence the viability of the technique and optimization of clinical outcomes.

## 2. Long-Term Changes to the Peritoneum and Related Consequences in PD

The peritoneal membrane is a structure that comprises mesothelial cells (an epithelial-like cell type) attached to a basement membrane. Underneath the basement membrane, there is the submesothelial layer composed of connective tissue, fibroblasts, and a dense vascularization. In PD, the peritoneum acts as an efficient semipermeable dialysis membrane when a hypertonic solution is introduced into the peritoneal cavity. Hypertonicity is generally obtained with glucose-based solutions, and thus the peritoneum is continuously exposed to unphysiologically high concentrations of glucose, resulting in cellular stress.

The rate of removal of metabolites, uremic toxins, electrolytes, and water from the systemic circulation is proportional to the vascular surface area in contact with PD fluid in the peritoneal cavity [10]. It has been shown that with ongoing dialysis, there is a continuous increase of submesothelial thickness and a particular vasculopathy. The vasculopathy is characterized by vessel-wall sclerosis and luminal thinning, as well as an increase in the number of blood vessels in the peritoneal tissues, and this correlates with UF failure [11]. In addition, the mesothelium undergoes progressive changes, starting with the loss of microvilli, cellular hypertrophy, and augmented vacuolation. Subsequently, mesothelial cells can also detach from the basement membrane, and this is accompanied by thickening of the submesothelial zone [12]. Mesothelial cells are highly metabolically active cells and are able to regulate peritoneal inflammation and the remodeling of the peritoneal tissue by secreting inflammation mediators, chemokines, growth factors, and components of the extracellular matrix (ECM) [13]. These histologic changes are associated with changes in peritoneal membrane function, such as a progressive increase in solute transport and a decrease in ultrafiltration capacity (8). It is notable that the development of peritoneal fibrosis is preceded by functional changes that alter water and solute permeability [14].

Overall, peritoneal fibrosis can be considered as the final step of peritoneal injury, and is regulated by several related processes: inflammation, angiogenesis, and epithelial-to-mesenchymal transition (EMT) [15]. In the peritoneum, the more correct designation of EMT is the mesothelial-to-mesenchymal transition (MMT). During this event, mesothelial cells lose their epithelial phenotype characterized by cell-to-cell contacts, cell-matrix interaction, and cell polarity, and acquire a mesenchymal phenotype. Mesothelial cells acquire the ability to migrate towards the submesothelium, where they contribute to the production of the extracellular matrix (ECM), thus resulting in fibrosis [16]. During MMT, mesothelial cells lose the expression of epithelial markers such as E-cadherin, cytokeratin, and zona occludens-1 (ZO-1), and begin to express the mesenchymal markers N-cadherin, vimentin, and α-smooth muscle actin (α-SMA) [17]. It has recently been demonstrated that the supraphysiological glucose concentrations currently used in PD solutions are able to activate MMT [18]. Thus, the use of glucose-sparing PD solutions could mitigate peritoneal fibrosis.

### 2.1. The Role of Growth Factors and Cytokynes

It has been shown that long-term exposure to high glucose concentrations causes changes in mesothelial membrane permeability and structure and, in particular, induces mesothelial cells to produce transforming growth factor-beta (TGF-beta) [19]. TGF-beta, which can also be produced in response to GDPs and AGEs [7], is a key fibrogenic factor involved in PD-associated peritoneal fibrosis [20], since it induces MMT via Smad-dependent and -independent pathways [21] while also activating inflammation and neoangiogenesis [7]. In particular, TGF-beta is responsible for peritoneal angiogenesis by directly inducing vascular endothelial growth factor (VEGF) and angiogenesis [22], especially in mesothelial cells undergoing MMT [23]. TGF-beta also produces an expansion of the submesothelial extracellular matrix, making the tissue hypoxic; this hypoxia drives a secondary angiogenic response [24]. Consequently, the control of TGF-beta production represents an interesting pharmacological strategy for the prevention of peritoneal fibrosis [25,26,27,28,29].

Peritoneal injury causes the activation of macrophages, neutrophils, and endothelial and mesothelial cells, which are the main sources of proinflammatory cytokines [30]. Similarly, high glucose concentrations in PD solutions result in a dose-dependent increase in the intraperitoneal production of IL-6 [31] and IL-1beta [32], which in turn enhances VEGF production and secretion, promoting microvascular permeability and angiogenesis [33]. High-throughput experiments have demonstrated that the secretion of profibrotic, proangiogenic, and proinflammatory cytokines such as TGF-beta, VEGF, and IL-6 [34,35,36,37] is associated with a decline in ultrafiltration and membrane protein loss [38].

### 2.2. The Role of Glycolysis, Glutaminolysis, and Fatty Acid Oxidation

The balance between rates of glycolysis, glutaminolysis, and fatty acid oxidation determines the deposition and breakdown of collagen and other ECM components that result in fibrosis [39]. This occurs as a result of the opposing effects of nonoxidative and oxidative pathways of ATP formation and the supply of amino acids to support the high rate of protein synthesis and the post-translational modifications required.

Hyperglycaemia results in the upregulation of TGF-beta and hypoxia-inducible factor 1 subunit alpha (HIF1α) expression [40]. Indeed, the transcription of TGF-beta is itself stimulated by HIF1α. The two co-ordinately act to increase glycolytic rate (TGF-beta) and inhibit the oxidation of the product of glycolysis, pyruvate, as HIF1α activates the expression of PDK1, which phosphorylates and inhibits pyruvate dehydrogenase (PDH). Glycolytic intermediates are important in the synthesis of amino acid substrates for collagen synthesis [41], whereas lactic acid release from myofibroblasts promotes lactylation of lysine residues in extracellular proteins, favouring the conversion of macrophages to an inflammatory phenotype [42]. Consequently, under the combined effects of increased TGF-beta and HIF1α expression, the elevated rate of glycolysis and diversion of pyruvate to lactic acid formation results in increased collagen synthesis, acidification, and proliferation of the ECM, which is accompanied by a lower rate of ECM degradation, essentially promoting fibrosis.

Another substrate that is increasingly utilized by cells that make the epithelial-mesenchymal transition to myofibroblasts is glutamine. This amino acid is itself important for collagen synthesis, and glutaminolysis gives rise to NH2 groups—which can be used in amino acid synthesis—as well as to another amino acid, glutamate. Conversion of glutamate to ketoglutarate (α-KG) provides a substrate for the generation of NADH and FADH2 through the oxidation of α-KG as a TCA cycle intermediate. These provide ATP through oxidative phosphorylation, which becomes increasingly important in the absence of substantial oxidation of pyruvate for this purpose in myofibroblasts, in a similar manner to the use of glutamine for ATP formation by neoplastic tissues [43]. However, the raised level of α-KG also stimulates the rate of prolyl hydroxylation [44], which is essential for collagen synthesis and stabilization in the ECM. Moreover, the succinate formed as a result of the partial oxidation of α-KG accumulates promotes the expression of HIF1α [45]. Thus, glutaminolysis is profibrotic.

Conversely, ECM degradation is favoured by mitochondrial oxidation of fatty acids. This is promoted by activation of the energy-sensing kinase AMPK, which, through its phosphorylation and inhibition of the acetyl-CoA carboxylases, lowers the concentration of the unique inhibitor—malonyl-CoA—of the rate-limiting enzyme of mitochondrial long-chain FA oxidation, carnitine palmitoyltransferase 1 [46]. However, under hyperglycaemic conditions, the activity of AMPK is diminished, resulting in the inhibition of fatty acid oxidation. Therefore, in fibrotic tissues, both pyruvate and fatty acid oxidation by mitochondria are inhibited. In the absence of a high rate of oxidation of fatty acids, long-chain acyl-CoA esters accumulate and promote the synthesis of complex lipids that are lipotoxic to fibroblasts and inhibit ECM degradation. Consequently, hyperglycaemia-induced inhibition of fatty acid oxidation helps to lower the rate of ECM degradation and promote fibrosis.

### 2.3. The Positive Role of L-Carnitine

The relationship between FAO and pyruvate oxidation is regulated primarily at the level of intramitochondrial acetyl-CoA (which activates PDK, thus inhibiting pyruvate oxidation) and cytosolic malonyl-CoA, which inhibits CPT1 and thus lowers FAO [47]. Importantly, carnitine—which is required for transport of long-chain FAs across the mitochondrial inner membrane for oxidation—once regenerated within the mitochondrial matrix also lowers intramitochondrial acetyl-CoA through the CAT-catalysed formation of acetyl-carnitine. The rapid efflux of acetyl-carnitine from the mitochondria favours increased pyruvate oxidation. This provides a possible pharmacological antifibrotic strategy, namely supplying tissues with supraphysiological concentrations of L-carnitine in the extracellular medium [48].

Raising the intracellular concentration of carnitine would shift the equilibrium catalysed by the intramitochondrial enzyme CAT towards acetyl-carnitine formation, thus lowering mitochondrial matrix acetyl-CoA and enhancing the release of the resulting acetyl-carnitine from the mitochondria in exchange for cytosolic carnitine. Such increases in intracellular carnitine would be difficult to achieve under normal physiological conditions, as dietary carnitine (given as oral supplements) is efficiently excreted by the kidney. However, it is possible to raise plasma concentrations of carnitine by many-fold in patients with end-stage kidney disease on peritoneal dialysis, in which residual kidney function is greatly decreased, through the use of carnitine-supplemented PD solutions [49].

Since PD patients experience fibrosis of the peritoneal membrane, such a strategy would be particularly appropriate to prevent fibrosis of the peritoneal membrane itself. Increased availability of carnitine to the cells that make up the peritoneal membrane would be anticipated to divert more pyruvate towards oxidation and away from lactate formation, thus decreasing the profibrotic profile of the myofibroblasts in the membrane.

### 2.4. Glycolytic, Fatty Acid, and Pyruvate Metabolism as Targets to Control Peritoneal Fibrosis

Alternative pharmacological strategies aimed at preventing/alleviating fibrosis could therefore be based on promoting fatty acid and/or pyruvate oxidation, or even by inhibiting glycolysis. In the former instance, the activity of CPT1 could either be increased directly pharmacologically; e.g., with compounds that, like C75, directly activate CPT1 [50]. Alternatively, the intracellular concentrations of malonyl-CoA in myofibroblasts could be lowered by the activation of the enzyme malonyl-CoA decarboxylase (MCD) [51]. Conversely, the activation of pyruvate oxidation could be achieved through the inhibition of PDK1 using dichloroacetate (DCA) [52]. Inhibition of this kinase would result in the activation of PDH and diversion of glycolytically produced pyruvate into the TCA cycle, rather than the formation of lactic acid and its release into, and acidification of, the ECM. Inhibition of glycolysis can be achieved pharmacologically through the use of 2-deoxyglucose, a glucose derivative that, once inside the cell, is phosphorylated by hexokinase 2 to 2-deoxyglucose-phosphate, which acts as a suicide inhibitor of hexokinase 2, and hence of glycolysis [53]. As already mentioned, TGF-beta1 is a key facilitator of the EMT transition by inducing a Warburg effect and switching cellular energy provision from oxidative phosphorylation to substrate-level phosphorylation through aerobic glycolysis [54]. Therefore, it is noteworthy that Si et al. have recently shown that by attenuating high glycolytic fluxes with 2-deoxyglucose, they could remarkably reduce TGF-beta1-induced profibrotic cellular phenotype in peritoneal mesothelial cells and peritoneal fibrosis in mice induced by a high peritoneal glucose load similar to that used in peritoneal dialysis [55]. It remains to be established whether the inhibition of glycolysis is a safer strategy compared to the diversion of pyruvate metabolism towards oxidative phosphorylation [56].

In summary, several metabolic mechanisms (glycolytic, fatty acid, and pyruvate metabolism), as well as extracellular factors (TGF-beta, VEGF, and inflammatory cytokines) and intracellular signalling (HIF-1α), are involved in mesothelial fibrosis, and their regulation should be considered in the development of new biocompatible PD solutions.

## 3. Strategies Devised to Improve the Biocompatibility of PD Solution

Strategies devised to reduce or eliminate PD-solution-associated toxicity without jeopardizing the patient’s health represent a major challenge in present-day PD therapy (Table 1).

The results obtained with the various strategies are reported in the following sections.

### 3.1. Neutral-pH, Low-GDP Solutions

The introduction of novel, glucose-based dialysates characterized by neutral- or physiological-pH and low-GDP fluids using multichamber bags was a major advance in the development of PD [57]. Use of these PD fluids containing lactate and/or bicarbonate as pH-buffer compounds is associated with a significant reduction of systemic GDP load and circulating AGE concentrations [3], which may have important benefits for the PD patient [58,59,60]. Better preservation of residual urine formation and kidney function has been reported in PD patients treated with these solutions [61], and some clinical observational studies even suggested improved patient survival rates [62]. Over a 24-month observation period, hydration status assessed by bioimpedance spectroscopy increased gradually and was significantly higher in patients treated with low-GDP fluids than in a control group treated with standard PD solutions, likely due to diminished peritoneal UF [63]. Moreover, despite several in vitro and experimental in vivo studies [3], recent findings suggest that improved biocompatibility of neutral-pH, low-GDP fluids cannot be assumed [64]. Indeed, peritoneal membrane biopsies in PD children treated with such dialysates showed early peritoneal inflammation, hypervascularization, fibroblast activation, and epithelial–mesenchymal transition, which affected PD membrane-transport function [65]. However, caution is needed before drawing any definitive conclusion. Sugiyama et al. [66] reported very recently that neutral-pH, low-GDP solutions are better at preserving the peritoneal endothelial glycocalyx compared to conventional acidic solutions during prolonged PD. Endothelial glycocalyx forms most of the surface layer on the luminal side of blood vessels, and is composed mainly of proteoglycans, glycoproteins, and glycolipids [67,68]. It may change dynamically in response to different pathophysiological stimuli, including inflammation and hyperglycaemia, affecting vascular permeability [69,70]. This was demonstrated by Sugiyama et al. [66], in which biopsies of the peritoneum were obtained from patients treated either with conventional acidic solutions or with neutral-pH, low-GDP solutions (*n* = 11 for each group) at the time of catheter removal. Loss of glycocalyx of the peritoneal endothelium was found in patients treated with conventional dialysate, and was associated with UF failure and severe vasculopathy. These results suggest that neutral-pH, low-GDP solutions could help to protect peritoneal vasculature [66]; further investigations are required.

### 3.2. Glucose-Free Approaches to Peritoneal Dialysis Solutions

Significant peritoneal damage is still observed with neutral-pH, low-GDP solutions, which suggests that the high glucose content in the dialysate (required for UF) is the main culprit of the peritoneal changes occurring over time in PD patients regardless of other possible causal factors [49]. The effects of such high glucose exposure include not only a detrimental role on the peritoneal membrane, but also many potential systemic metabolic side effects associated with chronic hyperglycaemia, including insulin resistance, new onset diabetes, and cardiovascular disease [71,72], due to intraperitoneal absorption of glucose from the dialysate. Patients with a higher peritoneal glucose absorption had an increased risk of 2-year cardiovascular mortality independent of other cardiometabolic risk factors [73].

Therefore, minimising the glucose-associated toxicity (glucose sparing) may be fundamental to improving the biocompatibility of PD solutions [49]. Several compounds have been used as alternatives to glucose, but only two osmotic agents are currently available in glucose-free dialysate for PD clinical practice: icodextrin and amino acids. However, it should be noted that such osmolytes can replace no more than 30–50% of the daily glucose dose [74], and can only be used in a single daily peritoneal exchange [75,76]. Moreover, no biopsies of peritoneal tissues have been obtained so far from patients maintained on icodextrin or amino acid solutions to provide direct insight into their impact on the peritoneal membrane [3].

***Icodextrin*** is a water-soluble glucose polymer derived from starch. It allows for a slow but sustained peritoneal UF, and is therefore indicated for use during a single long dwell per day [77,78]. Icodextrin is of particular value in anuric patients and in patients with fast peritoneal transport, and it improves patient UF without increasing the risk of adverse effects [79]. A recent systematic review and meta-analysis demonstrated that an icodextrin-containing PD solution is associated with fluid-handling benefits, such as improvement of peritoneal ultrafiltration and fewer episodes of fluid overload [80]. Indeed, the use of icodextrin-containing solutions in chronic heart failure patients who were refractory to conservative treatment proved to be a viable option leading to a better cardiac functional status, and lowered the number of hospital admissions [81,82]. Some studies have also shown that long-term utilization of icodextrin solution in ESKF may extend patient survival and PD viability [83,84].

A combined crystalloid (glucose) and colloid (icodextrin) PD solution has been used as a glucose-sparing strategy, with positive results. It increased peritoneal ultrafiltration while protecting the peritoneal membrane and diminishing the systemic consequences, namely systemic absorption of glucose in high and high-average transport APD patients. Freida et al. [85] evaluated the use of a glucose-sparing solution by replacing 7.5% icodextrin with a mixed crystalloid and colloid PD fluid (bimodal UF) during the long dwell in a group of APD patients, in an attempt to promote daytime UF and sodium removal while diminishing the glucose strength of the dialysate at night. They demonstrated that a bimodal solution based on the mixing of glucose (2.6%) and icodextrin (6.8%) achieved the double target of significantly improving UF and peritoneal sodium removal by exploring a new concept of glucose-sparing PD therapy [85].

In terms of biocompatibility, despite being glucose-free and with a low content of GDPs, conventional icodextrin solutions have a low pH, which can result in increased local and systemic inflammation [86,87]. A potential countermeasure may be the recent development of a neutral-pH icodextrin dialysate. This new icodextrin solution is delivered in two chambers, one containing icodextrin and the other containing electrolytes, with the two components being mixed just before use. Yamaguchi et al. [88] reported better viability of human mesothelial cells with the new two-chamber icodextrin solutions compared to the conventional, single-chamber one. More recently, the effects of such PD fluids were examined in an in vitro model involving cultured rat mesothelial cells [89]. An increase in α-smooth muscle actin, collagen type 1 and 3, and P21 mRNA expressions occurred when cells were incubated with the acidic conventional solution. The latter was also associated with inhibition of cell growth, induction of cell senescence, stimulation of epithelial–mesenchymal transition, and induction of fibrotic changes. All these unfavorable effects were not observed when mesothelial cells were cultured with the neutral-pH icodextrin solution [89]. The adverse effects of acidic icodextrin on mesothelial cells was attributed to low pH and a higher GDP content than the neutral icodextrin solution [88,89]. The development of a neutral pH dialysate represents an important advance in efforts to improve the biocompatibility of icodextrin-containing PD fluids.

The other commercially available glucose-free PD solution is based on the use of ***amino acids***. It has a pH of 6.7 and is free of GDPs. In addition, this approach offers the possibility of improving the nutritional status of some malnourished PD patients. Use of amino-acid-based PD fluids has been shown to increase muscle amino acid uptake [90] and improve anthropometric parameters, particularly those indicating muscle mass and fat stores, [91] while controlling urea levels [92]. The gluconeogenic potential of the amino acids should, however, be borne in mind for PD patients who may already be glucose-intolerant, insulin-resistant, or overtly diabetic.

The biocompatibility of PD solution containing amino acids remains uncertain [3]. Experimental studies in a rat model of PD showed that exposure to amino acid PD fluid was associated with a reduced peritoneal AGE deposition, lower levels of vascular endothelial growth factor, and a lower vessel density compared to treatment with standard glucose-based PD solutions [93]. However, in human peritoneal mesothelial cells cultured with amino-acid-containing PD dialysate, there was increased generation of nitric oxide [94], a finding which may have pathophysiological relevance [95].

As a glucose-sparing approach, a low-glucose regimen based on the use of amino acids, icodextrin, and dextrose was examined in two randomized studies in PD diabetic patients [96]. The intervention group had statistically lower glycated hemoglobin A1c at 6 months compared to the standard glucose–PD fluid group. Some improvements in triglycerides, VLDL, and apolipoprotein B were also observed in the glucose-sparing group [96], but high levels of carbamylate albumin (a marker of carbamylation load) was not reduced [97]. In the intervention group, however, a greater number of deaths and serious adverse events, including several related to extracellular fluid volume expansion, were reported [96]. While these results might have been influenced by lower concentrations of dextrose-based dialysis fluid in the pursuit of better metabolic outcomes, when more hypertonic dialysate was indicated to optimize ultrafiltration, they strongly emphasized the importance of close clinical monitoring of the patient’s fluid status when using any glucose-sparing strategy [49].

An interesting low-molecular-weight osmolyte that has been tested either alone or in combination with amino acids is ***glycerol*** (molecular weight 92 Da), a small three-carbon alcohol used as the backbone of triglyceride (TG) and phospholipids. As glycerol becomes available in the circulation, either after hydrolysis of dietary fat (TG), lipolysis of adipocyte stored TG, or glycerol-based PD solution, it is primarily metabolized by the liver due to the presence of aquaporin 9, which facilitates its transport into the hepatocyte, and a very active glycerol kinase that phosphorylates it to glycerol-3-phosphate [98]. The fate of liver glycerol-3-phosphate generated via the daily load of glycerol-based PD solution may raise some concerns—according to the nutritional state, glycerol-3-phosphate can be used for lipogenesis in the fed state or gluconeogenesis in the fasted state. In addition, the presence of insulin-resistance and/or diabetes, commonly present in PD patients, may further increase gluconeogenetic and liponeogenetic fluxes, leading to an increased CVD risk. Indeed, one of the first clinical trials in which glycerol was used as an osmotic agent instead of glucose, PD patients experienced a significant increase of plasma TG along with free glycerol, particularly those receiving glycerol-based PD solution at the highest concentration [99].

Van Biesen et al. used an alternative strategy to reduce the daily peritoneal exposure to glucose by formulating a PD solution with a mixture of 0.6% amino acids and 1.4% glycerol that was tested in a randomized, 3-month trial in nondiabetic CAPD patients [100]. The daily dialysis regimen consisted of two exchanges with amino acid/glycerol solution replacing two 2.27% glucose-based standard solutions (which were used in the control group), one exchange with icodextrin, and one exchange with a classic glucose solution. Clinical use of the new PD solution proved to be safe and well tolerated by patients, and was associated with an ultrafiltration capacity comparable with that of glucose, a significant reduction of glucose absorption, and increased dialysate levels of CA 125 (a potential marker of better biocompatibility) [100]. From the systemic standpoint, however, it remains to be established if a daily peritoneal glycerol load would further increase hepatic glucose and VLDL-TG production over time, particularly in insulin resistant and/or diabetic PD patients [98]. Smit et al. [101] switched 10 PD patients with UF failure from glucose-based dialysis solutions to a dialysis regime consisting of two to three exchanges (according to the patients’ needs) with a 2.5% glycerol-based dialysate, one exchange with a 1.1% amino acid-based dialysate, and one exchange with 7.5% icodextrin-containing dialysate for 3 months. Four patients were diagnosed with encapsulating peritoneal sclerosis (PS), proven by peritoneal biopsies. In the whole cohort, no statistically significant changes were observed for transport characteristics after 6 weeks or 3 months of glucose-free treatment, compared to the baseline levels [101]. However, after 6 weeks of glucose-free treatment, non-PS patients showed a significant increase in the transcapillary ultrafiltration rate and a decrease in the mass transfer area coefficient of creatinine. By contrast, no significant changes were found in patients suffering from PS. These results suggest that early withdrawal of glucose-based dialysis solutions, or at least a marked reduction in glucose exposure, may improve peritoneal function in PD patients with UF failure, but the identification of the patients who would benefit most warrants further studies [101]. In this regard, and in a broader sense to improve the clinical outcomes of PD, it is necessary to identify new biomarkers of the health of the peritoneal membrane in relation to dialytic prescription as tools in guiding personalized interventions in patients who are at risk of PD-related complications [102]. Proteomic analysis of PD dialysate might help to identify biomolecules that are indicative of the peritoneal health, peritoneal transport status, and ongoing pathological processes [103].

The biocompatibility profile of glycerol-containing PD solution was examined in a rat model. A bicarbonate/lactate-buffered solution with a mixture of osmotic agents (glycerol 1.4%, AA 0.5%, and dextrose 1.1%) (GLAD) was used daily for 16 weeks in a randomized study in a rat model of chronic renal failure [104]. As compared to 3.86% glucose-based solution, GLAD exposure was associated with a good preservation of peritoneal morphology in terms of the amount of fibrosis in the omentum (submesothelial, intersegmental, and perivascular areas) and the omental vessel density. Other authors examined the effects on peritoneal function in rats and the morphology of the peritoneal membrane after 20 weeks of peritoneal exposure to three different solutions: (i) a filter-sterilized, pyruvate-buffered solution with a combination of three osmotic agents (amino acids, glycerol, glucose: PYRAGG); (ii) a conventional heat-sterilized solution; or (iii) a filter-sterilized solution [105]. Filter sterilization avoided generation of GDPs. It is noteworthy that the PYRAGG solution was buffered with pyruvate, a key metabolite in the intersection in the network of metabolic pathways, replacing lactate. While peritoneal solute and fluid transport rates at 20 weeks were similar in all groups, PYRAGG solution proved to be more biocompatible than the other solutions, being associated with lowest number of omental vessels and less-pronounced peritoneal fibrosis [105], in keeping with the results of a previous study showing a 50% reduction in the number of peritoneal blood vessels by using a pyruvate-buffered dialysis solution as compared to a standard lactate-buffered fluid [106]. Any advantage of pyruvate compared to lactate in the absence of high glucose concentrations remains to be established, since aqueous solutions of pyruvate rapidly undergo an aldol-like condensation reaction to form 2-hydroxy-2-methyl-4-ketoglutarate (parapyruvate), a potent inhibitor of a key step in the Krebs cycle if taken up by the cells [107].

Other compounds that have been tested for a potential use in the PD solution as osmotic agents to replace glucose include taurine and hyperbranched polyglycerol, both still under experimental development.

***Taurine*** has a molecular weight of 125 Da and is a sulfonic beta-amino acid with high water stability that is present in high concentrations in mammalian cells, where it regulates osmotic balance and ion transport. Peritoneal transport and biocompatibility of a new PD solution containing taurine instead of glucose as the osmotic agent were examined in a rat PD model [108]. The new solution had a neutral pH and undetectable levels of GDPs. An approximately 3.5% taurine-based PD fluid achieved equivalent ultrafiltration to solutions containing 3.86% glucose. Also, taurine-containing PD solution proved to be more biocompatible than glucose-containing PD solutions with respect to the viability of the peritoneal membrane, inducing less mesothelial and fibroblast-like cell proliferation [108]. However, caution needs to be exercised if taurine is to be used as an osmotic agent in PD—when taurine is administered systemically, it undergoes very little metabolism, and most of it is excreted directly into the urine, so accumulation in the blood can be expected in patients with renal failure [108]. This has received support by an open nonrandomized trial in 10 chronic hemodialysis patients receiving oral taurine at a dosage of 100 mg/kg/day, comparable to that previously used in human clinical trials [109]. Neurological symptoms such as dizziness and non-rotatory vertigo appeared in two out of the first four patients, associated with a marked increase in plasma and muscle intracellular taurine levels. These symptoms rapidly disappeared after stopping taurine, but long-term risks of excessive accumulation cannot be ruled out and deserve further investigation.

***Hyperbranched polyglycerol*** is a polyether polymer synthesized by polymerization of glycidol that is hydrophilic, highly water-soluble, and chemically stable in aqueous solution. Du et al. [110] compared the effects over a 3-month period of a hyperbranched polyglycerol-containing PD (glucose-free, pH 7.4) vs. glucose-based PD solutions in a rat model of PD. While similar ultrafiltration and waste removal were achieved, the experimental solution was associated with a smaller change in both the structure and the angiogenesis of the PM and fewer cells expressing vascular endothelial growth factor, smooth muscle α-actin, and the macrophage marker MAC387 [110]. Moreover, transcriptome-based pathway analysis showed more inflammatory signaling pathways activated in the PM of the rat group treated with glucose-based dialysate than in the PM of the group exposed to hyperbranched polyglycerol [110]. A more recent study compared hyperbranched polyglycerol fluid to low-GDP and icodextrin PD fluid in a rat model (obese type 2 diabetic ZSF1 rats) of metabolic syndrome [111]. Metabolic syndrome is frequently observed in PD patients [112] and is associated with an increased all-cause and CVD mortality risk [113]. After the 3-month treatment period, PD fluid containing hyperbranched polyglycerol was found to better preserve the structures and function of the peritoneal membrane and kidneys, and to induce less systemic adverse effects on metabolism, immune response, and serum antioxidant capacity [111]. Results with this new PD solution are promising in terms of biocompatibility, although metabolism of polyglycerol and the possible consequences of plasma accumulation and tissue disposition with long-term use remain to be determined [3].

### 3.3. Addition of Membrane-Protective Compounds to the Peritoneal Dialysis Solution

The use of additives in PD dialysate to prevent, treat, or arrest bioincompatibility has been proposed as a strategy to attenuate the bioincompatible effects of the glucose-based PD solutions. Among potential additives, the use of glycosaminoglycans [114], heparin [115,116,117], citrate [118,119], peptides [120], or dipeptides [121,122] have initially been evaluated.

In one study, sixteen CAPD patients received ***sulodexide*** [114] for 30 days followed by a 30-day washout. For the night dwell (8–10 h), the patients were prescribed one 500 mL glucose-based PD solution bag with a glucose concentration of 111 mmol/L, containing 50 mg of sulodexide (a heparinoid formulation: 80% low-molecular-weight heparin and 20% low-molecular-weight dermatan sulphate). Sulodexide improved efficiency in these PD patients; specifically, permselectivity was improved, as shown by the parallel increases in urea and creatinine transport across the peritoneal membrane, as well as a reduction in protein (mostly albumin) losses.

The potential of the use of ***heparin*** during PD has been considered. Clinical experience with peritoneal rest and intermittent heparin administration has offered some limited success [115]. A couple of randomized trials with once-daily IP addition of low-molecular-weight heparin were carried out in Denmark (tinzaparin) and Spain (bemiparin) with different outcomes. Whereas the Danish study [116] concluded that long-term treatment of PD patients with IP tinzaparin reduced local and systemic concentrations of inflammatory markers, as well as improved UF and reduced small-solute peritoneal permeability, the Spanish study [117] did not support the daily IP addition of bemiparin, as it did not significantly improve UF or creatinine transport. However, they observed improved UF capacity in those patients with overt UF failure, in contrast to those patients in the control group. The different results in these two well-performed studies might be related to differences in study design: (a) both randomized, but one double-blinded, crossover, and with a placebo (isotonic saline) group, whereas the other was not blinded or crossover, and had a non-placebo control group; (b) one using heparin IP in the morning, a glucose-based PD solution, and a short dwell, with the other using heparin IP in an icodextrin-based PD solution and a long dwell; (c) one study with 21 and the other with 95 PD randomized patients; and (d) one lasting a total of 7 months and the other 6 months for each patient.

The apparent lack of effect of bemiparin on anti-FXa activity in the Spanish study may suggest noncompliance in some patients, although low peritoneal absorption is a plausible alternative explanation. On the other hand, the Danish study [116] presented a high incidence of peritonitis (seven cases during 102 patient-months). However, it is important to note that IP tinzaparin did not cause a higher incidence of peritonitis than treatment with IP placebo, leaving as probable cause the injection procedure with short needles (1.2 cm) due to technical difficulties in keeping sterile conditions during the procedure. One very important finding in both studies is that there was no increased risk of bleeding. Therefore, it seems that the potential of the use of heparin during PD remains relevant, and warrants careful review of the clinical trials performed so far. It may represent a new starting point for further application of heparin as an additive in PD.

In line with the application of heparin as an additive in PD, ***sodium citrate*** was chosen as a suitable candidate as an alternative inhibitor of complement [118]. The promising tinzaparin clinical trial results (long-term effects on increased UF and amelioration of the systemic inflammatory response in PD patients) published by Sjøland et al. [116] prompted Braide et al. [119] to initiate the first clinical study on the application of citrate as an additive in PD. Crossover design was used to evaluate sodium citrate and heparin-supplemented Gambrosol trio^®^ dialysate (2.5% glucose) in 28 stable outpatients from the PD unit. Comparisons were made between single dwells of each fluid. The addition of citrate (5 mM/L) to the PD solution significantly improved UF and small-solute clearances. The side effects of the use of citrate on calcium metabolism and acid–base balance were minimal.

***Carnosine*** and related compounds are associated with an effective regression in AGE formation in both long- and short-term exposures in vitro [120].

Werynski et al. [121] reported kinetic studies with ***dipeptide***-based PD solutions in 2001. Their results indicated that the hydrolysis rate of dipeptides in the peritoneal cavity is much lower than the dipeptide diffusive transport rate from the PD solution into blood, thereby providing more sustained UF when compared to amino-acid-containing PD solutions. Another interesting finding was that dipeptide-containing solutions may deliver amino acids across the epithelium with a smaller change in plasma amino acid concentrations [121].

Fifteen years later, Ferrantelli et al. [122] published a study using uremic rat and mice exposure models to evaluate the impact of the addition of pharmacological doses of ***alanyl-glutamine*** (Ala-Gln) to glucose-based PD solutions. The addition of Ala-Gln to the PD dialysate reduced peritoneal thickness, αSMA expression, and angiogenesis; prevented peritoneal extracellular matrix deposition; and attenuated the IL-17 pathway expression induced by glucose-based PD solutions. The results of two different dipeptide-based PD solution rat/mice studies, within 15 years of each other, have provided evidence that the use of PD solutions containing dipeptides may have a local protective effect (peritoneal membrane/cavity), as well as positive systemic effects (sustained UF, smaller changes in plasma amino acid levels), resulting in better biocompatibility [122]. More recently, PD fluid supplemented with Ala-Gln dipeptide (8 mM) could reduce PD-associated vasculopathy by reducing endothelial cellular damage, restoring the perturbed abundances of pathologically high levels of key proteins, and enabling protective processes [123].

Two early clinical trials performed by an Austrian study group on the addition of the dipeptide Ala-Gln to glucose-based PD solutions showed that it restored the stress response and improved immune competence of the peritoneal cells [124,125]. Vychytil et al. [126] performed the first randomized controlled trial with the addition of Ala-Gln to the PD solution, to assess the impact on biomarkers of peritoneal health. As expressed in the title of the study, this was an evaluation of a dipeptide as an additive to the PD solution and its impact on the local (peritoneal membrane) biocompatibility: a double-blinded, randomized crossover design, with stable PD patients (*n* = 50 randomized; eight Austrian PD centres) treated with Ala-Gln or placebo added to the PD solution for eight weeks. They concluded that Ala-Gln supplementation in PD solution improves biomarkers of peritoneal membrane integrity, immune competence, and systemic inflammation when compared to nonsupplemented PD solution with neutral pH and low-glucose degradation [126]. Consistent with the restoration of stress and immune processes observed in randomized clinical trials [124,125,126], targeted metabolomic profiling of PD dialysate suggested that the Ala-Gln-supplemented PD glucose solution had an antioxidant effect [127]. However, an adequately powered phase III trial is required to determine the impact of Ala-Gln as an additive on hard clinical outcomes.

Another potential PD solution additive to be explored in the prevention/arrest of PD solution bioincompatibility impacting the peritoneal membrane and cavity is ***molecular hydrogen*** (H2). Its unique biological action as an antioxidant ameliorates tissue injury [128]. Moreover, Nakayama et al. [129], in an elegant study using H2 dissolved in PD solutions, could preserve mesothelial cells and peritoneal membrane integrity in PD rats. H2 dissolved in water, given orally or by intraperitoneal administration, can suppress oxidative or inflammatory injury in various types of animal models by playing a role as modulator of the expression of various molecules, such as MAPK, MEK-1, NFκB, and caspase-3 and 12, and by upregulating Nrf-2, which could prevent oxidative injury and apoptosis [130]. Terawaki et al. [131] could reduce peritoneal and systemic oxidative stress without any side effects by single administration of H2-enriched dialysate. Hongtao et al. [132] recently showed that in a mouse model, after the induction of peritoneal fibrosis by a high-glucose solution, treatment with hydrogen-rich peritoneal dialysate exerted an antiperitoneal fibrotic effect, suggested to occur through the ROS/PTEN/AKT/mTOR pathway. These observations highlight the need for H2–PD clinical trials in the future.

### 3.4. Use of Metabolically Active Osmolytes (Osmo-Metabolites) in the Peritoneal Dialysis Solution

The wealth of data, information, and experience published with commercially available glucose-sparing PD solutions (icodextrin, amino acids) indicates that the future of PD depends largely on finding new osmotic agents, improving their biocompatibility and the fluid balance, but also, and no less important, its effect on the metabolism of the patient. The osmo-metabolic approach to PD solutions represents a novel tool to antagonize glucose-associated toxicity, which is based on the replacement of most of the glucose load with other osmolytes. Osmo-metabolites can be defined as those compounds that exhibit both osmotically and metabolically favourable properties [133,134]. While the above glucose-sparing strategies aimed at reducing patient glucose exposure hinge on the use of glucose-free PD fluid, the osmo-metabolic approach uses bioactive glucose-sparing osmolytes to both reduce intraperitoneal glucose load without compromising UF and mitigate the underlying systemic negative metabolic effects caused by the glucose load [49].

***L-carnitine*** (LC) and ***xylitol*** are two examples of osmo-metabolic agents. LC is a naturally occurring compound known to be essential for fatty acid oxidation in the mitochondria [135]. Xylitol, a five-carbon sugar alcohol (pentitol), is not only widely present in the plant kingdom, but is also a metabolic intermediate in mammals; up to 5 to 15 grams per day are formed in the human body [136]. Exogeneous or endogenous xylitol is further metabolized within the nonoxidative branch of the pentose monophosphate shunt, giving first rise to xylulose and then xylulose-5-phosphate, the latter being rapidly converted into glycolytic intermediates [136]. These two molecules share some common features, such as molecular weights similar to that of glucose (LC 161.2 Da, xylitol 151.2 Da), high water solubility and chemical stability in aqueous solutions, and osmotic properties, which render them suitable for use in PD fluids [49]. Studies on the biocompatibility of a PD solution containing carnitine or xylitol have shown a better profile than glucose-based solutions. Studies in mesothelial cells in vitro demonstrated that use of xylitol-containing PD solution, as compared to glucose-containing solution, is associated with an increase in the number of cells, no increase in interleukin 1 (expression of cellular injury), and lower formation of giant cells, as well as a higher concentration of indispensable surfactants for normal functioning peritoneum, such as phospholipids and phosphatidylcholine (Arduini A, Patent PCT/EP2006/060162). The good biocompatibility of LC-containing solution has been demonstrated in vitro in several experimental models, including rabbit mesothelial cells [137], human umbilical vein endothelial cells (HUVECs) [138], and murine fibroblast L929 [138], and in vivo in a rabbit model of PD [137].

Clinical studies demonstrated excellent tolerability and feasibility of xylitol [139] or L-carnitine [134] use in PD fluid. Bazzato and colleagues [139] treated six insulin-dependent diabetic patients on CAPD with D-xylitol as the sole osmotic agent in the PD solution (three daily exchanges of PD solution with xylitol 1.5% and one exchange with xylitol 3%). After a follow-up in 5 months, use of xylitol-containing PD fluid proved safe and maintained fluid balance, as indicated by peritoneal ultrafiltration, body weight, and mean arterial pressure [139]. Our trials on CAPD patients with L-carnitine-enriched dialysate demonstrated the effectiveness of L-carnitine as an osmolyte [138]. Moreover, L-carnitine-containing PD solution was associated with a better preservation of urine volume compared to controls (treated with standard glucose-based PD fluids) over a 4-month period [140]. This observation may be of clinical relevance, since urine output in PD patients is important in maintaining an appropriate volume control, one of the key goals in a PD prescription [141]. Recent practice recommendations by the International Society for Peritoneal Dialysis report that high-quality PD prescriptions should aim to achieve and maintain clinical euvolemia by taking into account residual kidney function and its preservation, such that both fluid removal from peritoneal UF and urine output are taken into consideration, and residual kidney function is not compromised [142].

Clinical use of xylitol- or LC-containing dialysate in CAPD patients has also been shown to induce favorable metabolic effects. The PD xylitol regimen described above [139] significantly improved the patients’ glycemic control, as indicated by the significant lowering of glycosylated hemoglobin and by a 50% reduction of required exogenous insulin. These positive effects on glucose homeostasis cannot be explained solely by the replacement of glucose (glucose-sparing action). Indeed, xylitol has a low glycemic index (a property that makes it more favorable than glucose if the caloric load needs to be better controlled, as in PD patients), it significantly inhibits hepatic glucose production [143], and it stimulates much less insulin secretion than does glucose [144]. As regards LC, use of the compound for 4 months in the PD fluid (2 grams per each of the 2/3 daily bags) of nondiabetic CAPD patients significantly ameliorated insulin resistance [140], a known cardiovascular risk factor [145,146]. Indeed, as shown by us and several other studies aimed at evaluating glucose homeostasis in both diabetic and nondiabetic animal models and humans, the achievement of safe LC supraphysiological levels significantly improves insulin sensitivity [48]. The mode of action of LC relates to its ability to modulate intra-mitochondrial acetyl-CoA levels (see above), a key metabolic intermediate able to affect both muscle glucose disposal and liver glucose production [48]. The precise mechanism involves the presence of a carnitine-dependent equilibrium reaction catalysed by carnitine acetyltransferase (CAT), the physiological role of which is to buffer the acetyl-CoA/free CoA ratio in mitochondria. As elevated acetyl-CoA levels may affect the activity of key enzymes involved in glucose metabolism in muscle (i.e., inhibition of pyruvate dehydrogenase) and liver (i.e., activation of pyruvate carboxylase), a supraphysiological increase of LC in mitochondria shifts the equilibrium of the CAT reaction towards acetyl-carnitine, limiting the rise of acetyl-CoA concentration and mitigating its unfavorable consequences on glucose homeostasis.

More recently, in the search for dialysates that minimize the negative effects of PD, we developed a new PD solution containing LC, xylitol, and low glucose, to achieve a favorable synergistic combination of the two osmo-metabolic agents. The biocompatibility of the experimental solution was first examined in cultured HUVECs obtained from the umbilical cords of either healthy or gestational diabetic mothers, and compared to a normal-pH, low-GDP PD solution [133]. The capillary endothelium represents the major barrier of the peritoneum to the transport of water and solutes. In both endothelial cell types, use of the experimental PD solution significantly improved cell viability, did not cause cytotoxicity, significantly reduced nitro-oxidative stress, and had none of the proinflammatory effects caused by hypertonic glucose-based PD solutions [133]. The presence of a low glucose concentration (27.7 mmol/L) in the experimental dialysate had no deleterious effects on the formulation of the PD solution, thus making it possible to take advantage of its UF ability.

We recently tested the biocompatibility of the new experimental PD solution by recreating in vitro a mesothelium-like structure, exploiting a cell line of human mesothelium grown to confluence on porous cell culture inserts previously coated with a layer of an extracellular matrix [147]. Using this experimental model, we compared the effects induced by several conventional glucose-based PD solutions, glucose-free solutions, and the novel dialysate formulation with xylitol and L-carnitine. Cells were exposed to the different PD solutions only on the apical side, to mimic what occurs during a PD dwell. This study provided compelling evidence that this novel formulation of PD solutions better preserves the integrity of the model mesothelial cell layer compared to conventional PD solutions. Cell viability proved to be highest for mesothelial cells exposed to the novel formulations. In addition, xylitol and L-carnitine better preserved the integrity of tight junctions in the mesothelial monolayers and prevented the drop in transepithelial electric resistance induced by conventional PD solutions. Of note, disruption of intercellular junctions and consequent loss of cell polarity in mesothelial cells trigger EMT, a process involving mesothelial cells’ transformation into fibroblast-like cells with increased migratory, invasive, and fibrogenic features [148]—see above. Moreover, analyzing a panel of 27 cytokines, chemokines, and growth factors, we found that the xylitol+L-carnitine PD solutions had a limited capacity to induce the activation of the inflammasome in mesothelial cells, thereby maintaining proper cellular homeostasis. Compared to conventional PD solutions, the xylitol+L-carnitine PD solutions induced a reduction of TNFα, interleukin-17A—regulated on activation T cell expressed and secreted (RANTES)—interferon-induced protein 10, VEGF, fibroblast growth factor, and platelet-derived growth factor BB. The local effect of L-carnitine on mesothelial cells, in particular the possibility that it might boost local acetylcholine production [149] and provide an additional anti-inflammatory effect [150], is an intriguing hypothesis that needs further investigation.

More recently, further in vitro data showed that the use of a PD solution containing carnitine and xylitol could prevent or mitigate the adverse effects produced by standard glucose-based PD solutions [151]. Results indicate that when compared to conventional glucose-based PD solution, the xylitol+L-carnitine formulation significantly preserves mesothelial cell viability and the mesothelial and endothelial phenotype, as well as their trans-epithelial resistance and permeability. In mesothelial and endothelial cells treated with the xylitol+L-carnitine solution, the gene expression of TGF-beta and SNAIL (its EMT-associated downstream transcription factor) was significantly lower than in cells treated with a glucose-based PD solution. Moreover, treatment of mesothelial and endothelial cells with carnitine–xylitol-containing solution did not activate MMT/EndoMT, suggesting an additional potential contribution to reducing peritoneal fibrosis. Lastly, a very mild angiogenic and inflammatory response with respect to the glucose-based solution was also observed [151].

A phase II, prospective, open, multicenter study to investigate the tolerability and the efficacy of osmo-metabolic agent-based PD solutions in CAPD patients is currently underway (NCT04001036). The study consists of three study periods (screening, intervention, and follow-up; each of four weeks) in two group of patients. Patients in group A were treated with a 2.5% glucose dialysate for the nocturnal exchange and, during the intervention period, received the experimental solution IPX15, while patients in group B received the experimental solution IPX07 replacing 1.5% glucose dialysate for two diurnal exchanges, maintaining icodextrin for the nocturnal exchange. Experimental bags differed in their xylitol content (IPX15 solution 98.6 mM, IPX07 solution 46 mM), but were otherwise identical in their composition, including L-carnitine 1.24 mM and glucose 27.7 mM.

Though the study has been greatly hampered and delayed by the COVID-19 pandemic and related implications, it is currently ongoing. Results obtained in the first cohort of patients completing the whole study period have been recently reported [152]. Use of the novel solutions proved well tolerated in all CAPD patients (group A, *n* = 6; group B, *n* = 4), and no adverse safety signals were observed. During the study, several efficacy parameters were assessed, including total weekly urea Kt/V, weekly total creatinine clearance, peritoneal equilibration test (which provides information about the transport characteristics of the peritoneal membrane), residual kidney function, daily diuresis, and daily peritoneal UF. The results indicate the noninferiority of the osmo-metabolic agent-based PD solutions compared to standard solutions as far as adequacy and peritoneal transport characteristics are concerned [152]. The completion of the study and the results of the planned ELIXIR trial (a 6-month randomized, controlled, parallel-group, international multicenter study; NCT03994471) will help to define the role of the proposed novel solutions in PD.

## 4. Conclusions

In PD, there is the unmet need for more biocompatible but efficacious dialysates to improve the clinical outcome and prolong the survival of the treatment. The ideal biocompatible PD solution should encompass three features: prolonged and sustained ultrafiltration capacity; preservation of the peritoneal membrane morphology and physiology; and, if its solutes are absorbed, they should not impact negatively on systemic metabolism and/or nutrition. By these criteria, little progress has been achieved in PD fluid technology over the last 30 years, and by the continued use of glucose-based fluids, PD treatment still confers major peritoneal and systemic toxicity [9]. Main advantages and disadvantages of PD solutions tested in vivo in PD patients are summarized in Table 2.

Initial clinical experience with some new approaches to improve the biocompatibility of fluids for PD is promising in terms of safety in the short- and mid-terms. More detailed and longer studies, some of which are already planned, are now warranted to establish whether the encouraging results obtained with the proposed novel PD solutions translate into important clinical outcomes.

## Figures and Tables

**Table 1:** Proposed strategies to improve the biocompatibility of standard peritoneal dialysis solution.

- Low/absent formation of GDP and neutral pH
Lactate buffer
Bicarbonate buffer
Lactate and bicarbonate buffer
- Replacement of glucose with other osmotic agent(s)
Icodextrin
Amino acids
Glycerol
Taurine
Hyperbranched polyglycerol
- Addition of cytoprotective agents
Sulodexide
Heparin
Sodium citrate
Carnosine
Alanyl-glutamine
Molecular hydrogen
- Use of osmo-metabolic agents
L-carnitine
Xylitol
L-carnitine and xylitol

**Table 2:** Main advantages and disadvantages of peritoneal dialysis solutions tested in vivo in dialysis patients.

	Glucose-Based Lactate Buffer	Biocompatible Glucose-Based Lactate and/or Bicarbonate Buffer	Icodextrin	Aminoacids	Glycerol and Aminoacids	Xylitol–Carnitine-Glucose	Glucose and Carnitine	Glucose and Alanyl-Glutamine	Glucose and Sulodexide
Glucose load	Max exposure	Max exposure	None	None	None	Less exposure	Exposure	Exposure	Exposure
Glucose sparing	No	No	Yes	Yes	Yes	Yes	No	No	No
GDP formation	High formation	Less formation	Less formation	None	None	Less formation	Yes	Yes	Yes
Potential advantage (systemic)	Nutritional	Nutritional	Volemia	Protein synthesis	Nutritional	Antidiabetic	Carnitine deficiency	Anti-inflammatory	Less protein loss
Potential advantage (peritoneal)	Osmotic	Osmotic and pH	Long-dwell UF	Osmotic	Osmotic	Osmotic, antifibrotic, and antiangiogenic	Osmotic and membrane preservation	Osmotic and membrane preservation	Osmotic and dialysis efficiency
Osmo-metabolic effects *	No	No	No	Yes	Yes	Yes	Yes	No	No

* Osmo-metabolic effects: PD solutions’ ingredients able to exhibit both osmotically and metabolically favorable properties.
